# Prognostic value of oxygen inhalation therapy for simple nocturnal hypoxemia in COPD: a meta-analysis

**DOI:** 10.3389/fphar.2023.1123945

**Published:** 2023-08-16

**Authors:** Ling Sun, Ya-Fei Chang, Yun-Fei Wang, Xian-Zhong Ran, Xiang-Rong Li, Quan-Xin Xie, Bin Ning

**Affiliations:** ^1^ Department of Respiratory and Critical Care Medicine, Fuyang Tumor Hospital, Fuyang, China; ^2^ Faculty of Forensic Medicine, Zhongshan School of Medicine, Sun Yat-Sen University, Guangzhou, China; ^3^ Department of Cardiology, The 901th Hospital of the Joint Logistics Support Force of the Chinese People’s Liberation Army, Hefei, China; ^4^ Department of Cardiology, Fuyang People's Hospital Affiliated to Anhui Medical University, Fuyang, China

**Keywords:** oxygen therapy, COPD, NOD, prognosis, meta analysis

## Abstract

**Objective:** The effect of oxygen therapy on the prognosis of chronic obstructive pulmonary disease (COPD) with nocturnal hypoxemia (NOD) has been controversial. Therefore, this study systematically evaluated the relevant literature and included it into randomized controlled studies for meta-analysis to evaluate the efficacy and prognosis.

**Methods:** We searched PubMed, EMBASE, web of science, Cochrane, China HowNet and Wanfang database for the literature on the prognosis of COPD patients with simple NOD from the establishment of the database to 30 June 2022. The outcome indicators were death and aggravation of the disease. The efficacy evaluation measures were pulmonary function and arterial blood gas results. The publication bias and heterogeneity of the included studies were evaluated.

**Results:** A total of 621 patients from 5 studies were included in this meta-analysis, and there was no publication bias in the included studies. The total mortality of long-term oxygen therapy (LTOT) in COPD patients with simple NOD in oxygen therapy group (RR = 1.04; 95% CI: 0.81–1.33, *p* = 0.77), mortality (RR = 0.87; 95% CI: 0.58–1.31, *p* = 0.50), risk of progression to LTOT events (RR = 1.07; 95% CI: 0.76–1.51, *p* = 0.71). PaO2 in patients with COPD and simple NOD in oxygen therapy group was higher than that in non-oxygen therapy group (mean difference (MD) = 13.47; 95% CI: 3.49–23.46, *p* = 0.008), the decrease of PaCO2 level was not statistically significant (MD = −10.05; 95% CI: −26.36-6.27, *p* = 0.23).

**Conclusion:** Oxygen therapy can improve the prognosis of blood oxygen partial pressure in COPD patients with simple NOD, but oxygen therapy has no significant effect on the survival rate, controlling the progression of the disease to LTOT and reducing the partial pressure of carbon dioxide.

## 1 Introdiction

Chronic obstructive pulmonary disease (COPD) is a common heterogeneous disease that can be prevented and treated. It is characterized by consistent respiratory symptoms and air flow limitation. It is usually caused by airway and/or alveolar abnormalities caused by obvious exposure to toxic particles or gases. Complications can increase the disability rate and mortality of COPD (Contreras-Garza et al., 2019). Its incidence rate continues to rise, and it is estimated that more than 5.4 million people will die of COPD and related diseases every year by 2060 (Global strategy for the diagnosis, 2015). The course of COPD is long, the condition is progressive, and the treatment effect of late COPD is poor, which seriously affects the quality of life of patients. Oxygen therapy is an important means to treat COPD patients with chronic persistent hypoxia symptoms and improve their prognosis (Al-Faqawi et al., 2018).

With the development of oxygen therapy research, non-invasive ventilation, nasal high flow oxygen inhalation and other oxygen therapy methods are applied in the treatment of COPD. Long term oxygen therapy can improve the survival rate of patients with COPD hypoxemia (PaO2<60 mmHg); the subjects of these studies are mostly patients with daytime hypoxemia. However, for patients with COPD who do not meet the criteria of long-term oxygen therapy, especially for patients whose PaO2 is not significantly reduced during the day but whose arterial oxygen saturation is decreased at night, less attention has been paid. FletcherDonner et al. (1992) showed that the mortality of COPD patients with PaO2 above 60 mmHg during the day but with simple nocturnal hypoxia destruction (NOD) increased significantly, which may be related to pulmonary hypertension and arrhythmia caused by insufficient alveolar ventilation or poor pulmonary ventilation perfusion during sleep (Sibila et al., 2014). In addition, whether oxygen therapy can improve the long-term survival rate of COPD patients with simple NOD is inconsistent with other outcomes. Chaouat et al. (1999) found that there was no significant difference in pulmonary artery pressure after 2 years between nocturnal oxygen therapy and non-oxygen therapy, and nocturnal oxygen therapy did not delay the time when patients began to use long-term oxygen therapy (LTOT). Wang et al. (1997) found that home oxygen therapy could significantly improve the survival rate of patients with daytime hypoxemia, but did not improve the survival rate of COPD patients with simple NOD. Tan et al. (2019) showed that the oxygen concentration was adjusted to 3–5 L/min according to the degree of hypoxia in COPD patients at different periods of night. As a result, the PaO2 of patients was improved, and the effect of oxygen therapy was better than that of continuous low flow oxygen inhalation. Fletcher et al. (1992) pointed out that nocturnal oxygen therapy (NOT) can improve the pulmonary artery pressure of patients with simple NOD in COPD, but the sample size was very small in the study, which affected the reliability of the study results to a certain extent.

Therefore, whether COPD patients with simple NOD need oxygen therapy, what kind of oxygen therapy measures should be taken, LTOT or NOT, and whether the effect of oxygen therapy can improve the prognosis of this population has been controversial. Therefore, this study evaluated the comprehensive efficacy and clinical prognosis of oxygen therapy and its measures in patients with simple NOD of COPD by systematic evaluation of relevant literature and meta-analysis of randomized controlled studies.

## 2 Materials and methods

The inclusion and exclusion criteria were in accordance with the Cochrane Handbook for Systematic Reviews manual (Higgins and Green, 2011).

### 2.1 Inclusion criteria and exclusion criteria

The inclusion criteria for this study are as follows:

1) Clinical study of oxygen therapy in patients with COPD nocturnal hypoxemia;
2) The end point of the study should include at least one of the following items: death, aggravation and readmission; The evaluation indexes of curative effect were pulmonary function and arterial blood gas;
3) Type of paper: Randomized, controlled study on oxygen therapy of COPD nocturnal hypoxemia;
4) The criterion of hypoxemia was that SaO2 < 90% time (SIT 90%) ≥ 30% of total sleep time.

The exclusion criteria for this study are as follows:

1) animal experiment;
2) Overview, commentary, case report, letter type literature, etc;
3) Repeated published literature;
4) Literature where the statistical method is not clear or the original data cannot be obtained.

### 2.2 Search strategy

This study was registered in PROSPERO (CRD 42022367613). We searched PubMed, Embase, Web of Science, Cochrane, CNKI, and Wanfang database from the beginning of the database to 30 June 2022. Use the keyword search strategy to find all the literature about “the effect of oxygen therapy on nocturnal hypoxemia of chronic obstructive pulmonary disease” (to select only the randomized controlled trial treatment of nocturnal oxygen therapy for COPD patients with simple nocturnal hypoxemia (that is, those who do not meet the long-term oxygen inhalation conditions). The search words are “chronic obstructive pulmonary disease” or “COPD”, “Hypoxemia”, “Oxygen therapy” or “Oxygen inhalation” “Efficacy” or “Prognosis”. The two authors (Ling Sun and Ya-Fei Chang) independently searched and screened the literature, and negotiated with the third author (Quan-Xin Xie) to solve problems encountered.

### 2.3 Document quality evaluation and data extraction

We conducted data extraction and quality assessment by using the preferred reporting item (PRISMA) method of systematic evaluation and meta-analysis (MoherShamseer et al., 2015; Shamseer et al., 2015). Two authors (Ling Sun and Ya-Fei Chang) independently extracted the following data from eligible studies: the name of the first author, the year of publication, the source of the study object, the sample size, the basic characteristics of the subjects, the oxygen therapy program, the follow-up time, evaluation indicators, the study endpoint and its RR, 95% CI, and confounding factors. If RR and 95% CI are not directly provided in the original literature, we will contact the original author to obtain the required information. Two evaluators independently evaluated the quality of the literature included in the study by using the Newcastle Ottawa Scale scoring scale. Any ambiguous problems will be solved through discussion by all authors.

### 2.4 Statistical analysis

The quality of the included study was assessed by the Cochrane Collaboration’s tool for Risk of bias. All cause death, deterioration of disease, re-hospitalization events, pulmonary function and arterial blood gas analysis results were counted. We used the inverse variance method to calculate Ln RR and standard error from the value of the natural logarithm of each study RR. We used Review Manager 5.3 software (The Cochrane Collaboration, Oxford, United Kingdom) for data analysis. When I^2^ ≥ 50%, it indicates that there is statistical heterogeneity in the study, and a random effect model is used; Otherwise, select the fixed effect model. *p* < 0.05 was statistically significant.

## 3 Result

### 3.1 Literature search results

**FIGURE 1:**
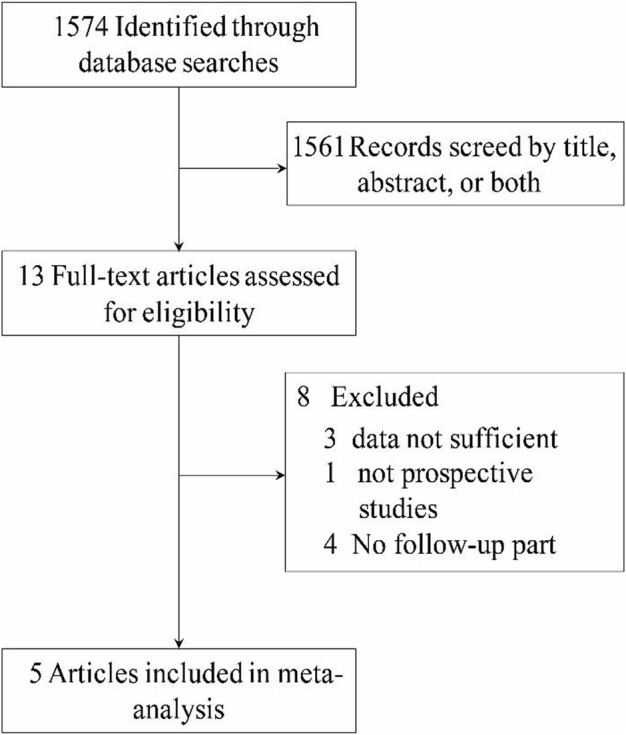
PRISMA flow diagram for the identification of studies included in the meta-analysis.

According to the retrieval criteria established in this study, we retrieved 1,574 articles in total. By reading the title and abstract, we preliminarily excluded 1,561 articles. Eight studies were excluded again by reading the text of the article: the data of three studies could not be combined for analysis or lacked extractable data, four studies did not follow up the subjects, and one study did not have a control group for its design type. Finally, five studies were included (Fletcher et al., 1992; Chaouat et al., 1999; Li et al., 2014; Xu et al., 2015; Yves et al., 2020). The process and results of retrieving articles are shown in Figure 1.

### 3.2 Characteristics of the included studies

**FIGURE 2:**
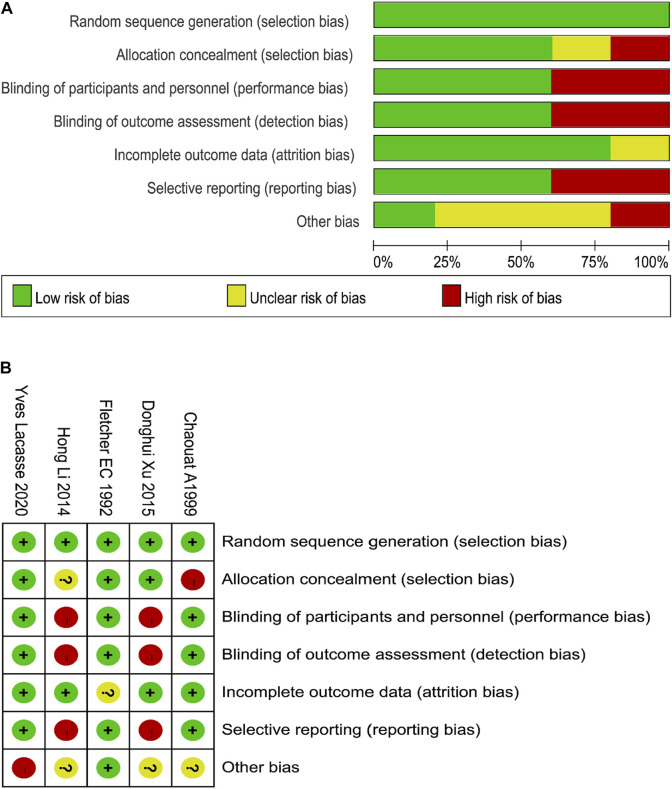
Document quality evaluation **(A** and **B)**.

The five studies included 621 participants, 312 in the oxygen therapy group and 309 in the control group. Two studies were from Asian populations, two from Europe and one from the United States. The follow-up time in different studies ranged from 2 to 4 years. The general characteristics and article quality evaluation of the included literature are shown in Table 1. In general, the quality of the included studies was good, a flow chart of the quality assessment of the studies is shown in Figures 2A and B.

**TABLE 1:** Patient demographics, baseline and study characteristics.

Author	Year	N	Design	Type of disease	Country	Outcome/evaluation index	Age, year	Follow up time	Intervention measures
Yves Lacasse	2020	A:123; B:120	Randomized, double blind, placebo-controlled	COPD Simple NOD	Spain Canada	Death or progression to LTOT and pulmonary hemodynamics	A:69 ± 8; B:69 ± 9	3–4 years	room air, 2L/min-4L/min
Fletcher EC	1992	A:19; B:19	Double blind, placebo-controlled, Randomized	COPD Simple NOD	United States	Death or progression to LTOT and pulmonary hemodynamics	63 ± 6	3 years	Nocturnal oxygen or room air, 3L/min
Chaouat A	1999	A:41; B:35	Double blind, placebo-controlled, Randomized, Open Label	COPD Simple NOD	France	Death or progression to LTOT、pulmonary hemodynamic indexes	A:63 ± 8; B:64 ± 6	35.1 month	Nocturnal oxygen therapy for 8–10 h a night
Donghui Xu	2015	A:60; B:66	Randomized controlled	COPD Simple NOD	China	Pulmonary function, arterial blood gas index, sleep structure	A:52–82; B:49–82	2 years	Not detailed
Hong Li	2014	A:69; B:69	Randomized controlled	COPD Simple NOD	China	Pulmonary function, arterial blood gas index, times of acute attack, times of hospitalization	A:56.3 ± 4.1; B:50.1 ± 6.7	3 years	Bilateral nasal catheter oxygen inhalation (2–4L/min), daily oxygen inhalation time >15 h

A, oxygen therapy group; B, non oxygen therapy group; NOD, nocturnal hypoxemia; COPD, chronic obstructive pulmonary disease.

### 3.3 Evaluation of oxygen therapy and different outcome indicators

#### 3.3.1 Total case fatality rate of LTOT

**FIGURE 3:**
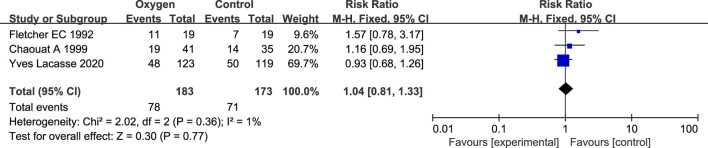
Overall case fatality rate results of COPD oxygen therapy and LTOT.

There were 3 studies on the combined outcome of COPD oxygen therapy and LTOT (Fletcher et al., 1992; Chaouat et al., 1999; Yves et al., 2020) (Table 2). There were 357 patients, including 183 patients in the oxygen therapy group and 174 patients in the control group. There was no statistical heterogeneity between the studies (*p* = 0.36, I^2^ = 0%), so the fixed effect model was used for analysis. Meta analysis results showed that there was no statistical difference in the total mortality of LTOT between the oxygen therapy group and the non-oxygen therapy group in COPD patients with simple NOD (RR = 1.04; 95% CI: 0.81–1.33, *p* = 0.77) (Figure 3).

**TABLE 2:** All prognostic/outcome indicators included in the study.

Author	Year	LTOT mortality	Death	Progress to LTOT	PaO2/mmHg	PaCO2/mmHg	SaO2/%
Yves Lacasse	2020	A:48/123; B:50/119	A:21/123; B:23/119	A:33/123; B:34/119	—	—	—
Fletcher EC	1992	A:11/19; B:7/19	A:5/19; B:6/19	A:6/19; B:1/19	—	—	—
Chaouat A	1999	A:19/41; B:14/35	A:9/41; B:9/35	A:12/41; B:10/35	—	—	—
Donghui Xu	2015	—	—	—	A:75.16 ± 5.98; B:56.59 ± 6.12	A:50.14 ± 4.29; B:68.51 ± 6.01	—
Hong Li	2014	—	—	—	A:68.27 ± 6.42; B:59.89 ± 6.12	A:45.08 ± 5.74; B:46.8 ± 5.37	A:92.88 ± 2.66; B:88.98 ± 2.96

A, Oxygen therapy group; B, Non oxygen therapy group; LTOT, long-term oxygen therapy.

#### 3.3.2 Mortality

**FIGURE 4:**
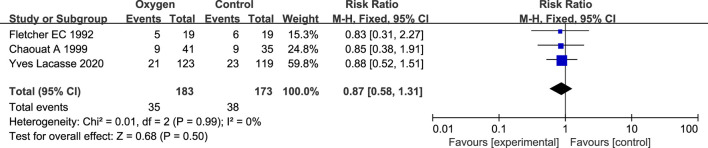
Results of COPD oxygen therapy and mortality.

There are 2 studies related to oxygen therapy and mortality in COPD (Fletcher et al., 1992; Chaouat et al., 1999; Yves et al., 2020) (Table 2). There were 219 patients, including 164 in the oxygen therapy group and 155 in the control group. There was no statistical heterogeneity between the studies (*p* = 0.99, I^2^ = 0%), so the fixed effect model was used for analysis. Meta analysis results showed that there was no statistical difference between the oxygen therapy group and the non-oxygen therapy group in the death risk of COPD patients with simple NOD (RR = 0.87; 95% CI: 0.58–1.31, *p* = 0.50) (Figure 4).

#### 3.3.3 Progress to LTOT

**FIGURE 5:**
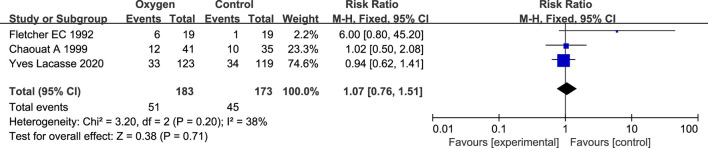
Research results of oxygen therapy and progress to LTOT.

There are 3 relevant studies on oxygen therapy and progress to LTOT (Fletcher et al., 1992; Chaouat et al., 1999; Yves et al., 2020) (Table 2). There were 357 patients, including 183 patients in the oxygen therapy group and 174 patients in the control group. There was no statistical heterogeneity between the studies (*p* = 0.20, I^2^ = 37%), so the fixed effect model was used for analysis. Meta analysis results showed that there was no statistical difference between the oxygen therapy group and the non-oxygen therapy group in the risk of LTOT events in COPD patients with simple NOD (RR = 1.07; 95% CI: 0.76–1.51, *p* = 0.71) (Figure 5).

#### 3.3.4 PaO2

**FIGURE 6:**
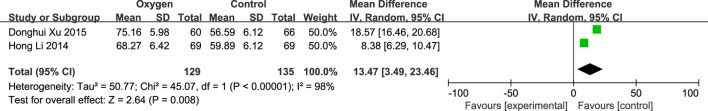
Research results of oxygen therapy and PaO_2_.

There are 3 relevant studies on oxygen therapy and PaO2 (Li et al., 2014; Xu et al., 2015) (Table 2). There were 264 patients, including 129 in the oxygen therapy group and 135 in the control group. There was statistical heterogeneity between the studies (*p* < 0.00001, I^2^ = 98%), so the random effect model was used to analyze the heterogeneity. The possible sources of heterogeneity were: the average age and age distribution of the subjects were different, the severity of the disease was inconsistent, and the statistical methods were different. Meta analysis results showed that PaO2 in patients with COPD simple NOD in the oxygen therapy group was significantly higher than that in the non-oxygen therapy group (mean difference, MD) = 13.47; 95% CI: 3.49–23.46, *p* = 0.008) (Figure 6).

#### 3.3.5 PaCO2

**FIGURE 7:**
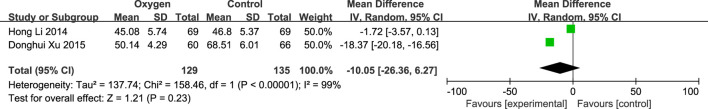
Research on oxygen therapy and PaCO_2_.

There are 3 relevant studies on oxygen therapy and PaCO2 (Li et al., 2014; Xu et al., 2015). There were 264 patients, including 129 in the oxygen therapy group and 135 in the control group. There is statistical heterogeneity between the studies (*p* < 0.00001, I^2^ = 99%), so the random effect model is used to analyze. The possible sources of heterogeneity are: the average age and age distribution of the subjects are different, the severity of the disease is inconsistent, and the statistical methods are different. Meta analysis results showed that there was no statistical difference between the oxygen therapy group and the non-oxygen therapy group in PaCO2 of COPD patients with simple NOD (MD = −10.05; 95% CI: −26.36–6.27, *p* = 0.23) (Figure 7).

#### 3.3.6 Publication bias analysis

The funnel diagram of the prognosis study of oxygen therapy and COPD hypoxemia is shown in Supplementary Figure S1. The funnel chart is symmetrical at the top and bottom, indicating that there is no publication bias in the included studies.

## 4 Discussion

In this research, we reviewed oxygen therapy and prognostic studies in patients with COPD with simple nocturnal hypoxemiasy stematically, concluding with an analysis of five prospective studies. Our results show that oxygen therapy improves the prognosis of COPD patients with nocturnal hypoxemia alone, but does not improve survival, control the progression of the disease to LTOT, or reduce the partial pressure of carbon dioxide. This study confirmed the results of the study on the prognosis of patients with oxygen therapy and COPD with pure nocturnal hypoxemia. There was no significant difference in the total mortality, LTOT mortality, and LTOT prognosis between the oxygen therapy group and the non-oxygen therapy group. Similar to the results of this study, the meta-analysis of Cranston et al. (2005) found that continuous oxygen therapy (at least 15 h a day) did not seem to improve the survival rate of patients with COPD with simple NOD. The difference is that Cranston et al. (2005) did not analyze the mortality and prognosis of LTOT. In the study by Yves et al. (2020), the endpoints were total mortality, LTOT mortality and progression to LTOT. The results showed that the above outcomes could not be improved by oxygen therapy. The study by Yves et al. (2020) was from multiple centers, with a more complete study protocol and a larger sample size, so the level of evidence was higher, and the results of the meta-analysis of this study with the results of the above-mentioned outcome indicators were similar. This study found that oxygen therapy can improve the PaO2 prognosis of patients with COPD with simple NOD, but there was no significant difference between the two groups at the end of follow-up. Xu et al. (2015) showed that the number of acute attacks, pulmonary function and arterial blood gas of patients in the oxygen therapy group were significantly improved compared with those in the non-oxygen therapy group. Li et al. (2014) found that oxygen therapy can improve PaO2, SaO2 and sleep disorders of COPD patients with simple NOD, and delay the progress of lung function through 3-year follow-up. According to Zhang and Gu. (1998), pressure support ventilation with biphasic positive pressure mask has obvious effect on improving sleep and respiratory disorder at night and correcting hypoxemia in patients with COPD. However, the study population was not included in this meta-analysis because it was an acute exacerbation population and the patients were not followed up. According to Wang et al. (1997), nocturnal oxygen therapy can correct nocturnal hypoxemia in most patients with chronic obstructive pulmonary disease, and noninvasive continuous positive pressure ventilation can achieve good results in patients with poorer outcomes. In addition, the study population was also an acute exacerbation population, and patients were not followed up and therefore were not included in this meta-analysis.

Heterogeneity analysis: In Xu et al. (2015) study, there are only upper and lower limits for the age of the subjects, and there is no average age or median age, so it is impossible to judge the age group and distribution of the subjects. In the study of Li et al. (2014), the age of the subjects was mean ± standard deviation. Xu et al. (2015) listed the partial pressure of oxygen and carbon dioxide of the subjects before treatment, while Li et al. (2014) did not mention the level of partial pressure of oxygen and carbon dioxide of the subjects before treatment. The aforementioned selection bias or statistical analysis differences may lead to statistical heterogeneity included in the study. Furthermore, the time and duration of oxygen therapy, the selection of oxygen therapy methods, the measures for evaluating the efficacy, and the oxygen therapy methods selected under different conditions are also different. The subjects came from different regions and the duration of follow-up was different.

Limitations: in various studies, the average age, age distribution, degree of nocturnal hypoxemia, follow-up time, complications, detection methods, *etc.*, of the subjects are different, which may lead to heterogeneity between different studies. The original studies included may have potential confounding factors. Although some studies use multivariate analysis to reduce confounding bias, the limitations and shortcomings of observational study cannot avoid confounding bias.

## 5 Conclusion

In conclusion, oxygen therapy can improve the prognosis of COPD patients with simple NOD, but oxygen therapy has no significant effect on the survival rate of patients, control the disease progression to LTOT, and reduce the partial pressure of carbon dioxide. Therefore, a larger sample of randomized controlled study is needed to conduct stratified analysis on patients with COPD with simple NOD, and to screen potential populations that may benefit from oxygen therapy.

## Data Availability

The original contribution presented in the study are included in the article/Supplementary Material, further inquiries can be directed to the corresponding author.

## Abbreviations

CI, confidence interval; COPD, chronic obstructive pulmonary disease; HR, provided hazard ratio; LTOT, long-term oxygen therapy; MD, mean difference; NOD, nocturnal hypoxemia; RR, Relative risk.
