# Supplementary material for: Prognostic value of oxygen inhalation therapy for simple nocturnal hypoxemia in COPD: a meta-analysis
Source: Front Pharmacol. 2023 Aug 16;14:1123945. doi: 10.3389/fphar.2023.1123945 (PMC10469590; doi:10.3389/fphar.2023.1123945)
Supplement: Supplementary file 1 [file DataSheet1.pdf]

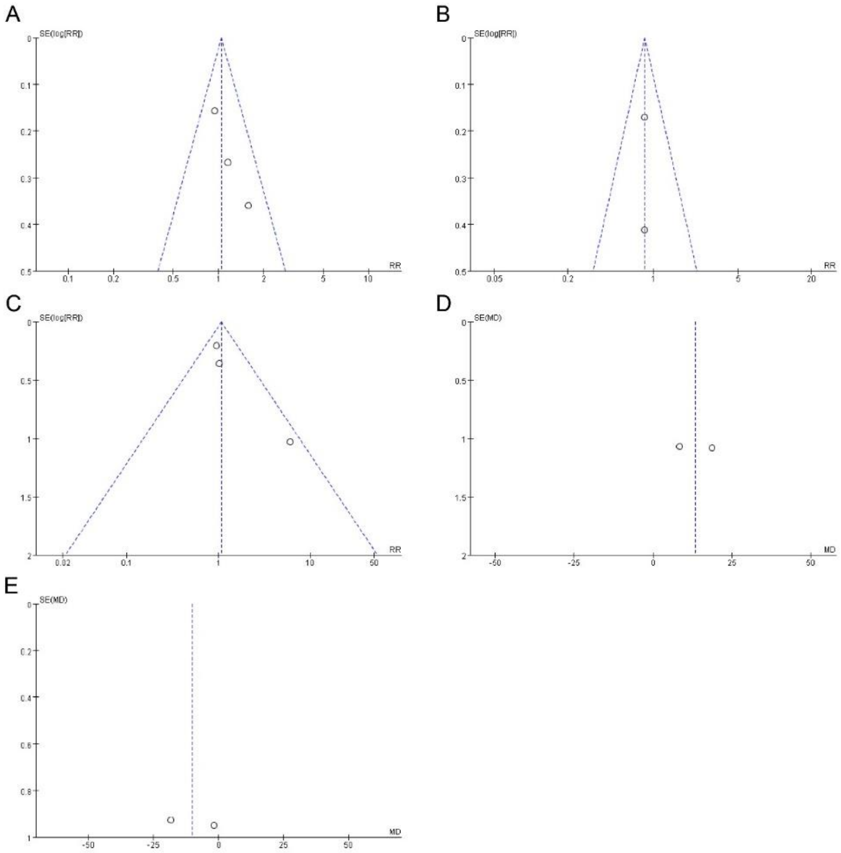

Figure S1. Funnel diagram of prognosis (A. Total case fatality rate of LTOT, B. Mortality, C. Progress to LTOT, D.PaO2, E. PaCO2) study of oxygen therapy and COPD hypoxemia.
